# "Are you gonna publish that?" Peer-reviewed publication outcomes of doctoral dissertations in psychology

**DOI:** 10.1371/journal.pone.0192219

**Published:** 2018-02-14

**Authors:** Spencer C. Evans, Christina M. Amaro, Robyn Herbert, Jennifer B. Blossom, Michael C. Roberts

**Affiliations:** 1 Clinical Child Psychology Program, University of Kansas, Lawrence, KS, United States of America; 2 Department of Psychology, Harvard University, Cambridge, MA, United States of America; 3 Department of Psychology, Washington State University, Pullman, WA, United States of America; 4 Department of Psychiatry & Behavioral Sciences, University of Washington School of Medicine, Seattle, WA, United States of America; Max Planck Society, GERMANY

## Abstract

If a doctoral dissertation represents an original investigation that makes a contribution to one’s field, then dissertation research could, and arguably should, be disseminated into the scientific literature. However, the extent and nature of dissertation publication remains largely unknown within psychology. The present study investigated the peer-reviewed publication outcomes of psychology dissertation research in the United States. Additionally, we examined publication lag, scientific impact, and variations across subfields. To investigate these questions, we first drew a stratified random cohort sample of 910 psychology Ph.D. dissertations from ProQuest Dissertations & Theses. Next, we conducted comprehensive literature searches for peer-reviewed journal articles derived from these dissertations published 0–7 years thereafter. Published dissertation articles were coded for their bibliographic details, citation rates, and journal impact metrics. Results showed that only one-quarter (25.6% [95% CI: 23.0, 28.4]) of dissertations were ultimately published in peer-reviewed journals, with significant variations across subfields (range: 10.1 to 59.4%). Rates of dissertation publication were lower in professional/applied subfields (e.g., clinical, counseling) compared to research/academic subfields (e.g., experimental, cognitive). When dissertations were published, however, they often appeared in influential journals (e.g., Thomson Reuters Impact Factor *M* = 2.84 [2.45, 3.23], 5-year Impact Factor *M* = 3.49 [3.07, 3.90]) and were cited numerous times (Web of Science citations per year *M* = 3.65 [2.88, 4.42]). Publication typically occurred within 2–3 years after the dissertation year. Overall, these results indicate that the large majority of Ph.D. dissertation research in psychology does not get disseminated into the peer-reviewed literature. The non-publication of dissertation research appears to be a systemic problem affecting both research and training in psychology. Efforts to improve the quality and “publishability” of doctoral dissertation research could benefit psychological science on multiple fronts.

## Introduction

The doctoral dissertation—a defining component of the *Doctor of Philosophy* (Ph.D.) degree—is an original research study that meets the scientific, professional, and ethical standards of its discipline and advances a body of knowledge [1]. From this definition it follows that most dissertations could, and arguably should, be published in the peer-reviewed scientific literature [1–2]. For example, research participants typically volunteer their time and effort for the purposes of generating new knowledge of potential benefit; therefore, to breach this contract by not attempting to disseminate one’s findings is to violate the ethical standards of psychology [3] and human subjects research [2,4]. The nonpublication of dissertation research can also be detrimental to the advancement of scientific knowledge in other ways. Researchers may unwittingly and unnecessarily duplicate efforts from doctoral research when conducting empirical studies, or draw biased conclusions in meta-analytic and systematic reviews that often deliberately exclude dissertations. Many dissertations go unpublished due to nonsignificant and complicated results, exacerbating the “file drawer” problem [5–6]. Indeed, unpublished dissertations are rarely if ever cited [7–8].

The problem of dissertation non-publication is of critical importance in psychology. Some evidence [9] suggests that unpublished dissertations can play a key role in alleviating file drawer bias and reproducibility concerns in psychological science [10]. More broadly, the field of psychology—given its unique strengths, breadth, and diversity—poses a useful case study for examining dissertation nonpublication in the social, behavioral, and health sciences. Like other scientific disciplines, many Ph.D. graduates in psychology may be motivated to revise and submit their dissertations for publication for the usual reasons offered by academic and research careers. However, other new psychologists might not pursue this goal for a variety of reasons. Those in professional and applied subfields may commit most or all of their working time to non-research activities (e.g., professional practice, clinical training) and have little incentive to seek publication. Even those in more research-oriented subfields increasingly take non-research positions (e.g., industry, consultation, teaching, policy work) or other career paths which do not incentivize publications. Negative graduate school experiences, alternative career pursuits, and personal or family matters can all be additional factors that may decrease the likelihood of publication. Moreover, it is typically a challenging and time-consuming task to revise a lengthy document for submission as one or more journal articles. Still, all individuals holding a Ph.D. in psychology have (in theory) produced an original research study of scientific value, which should (again, in theory) be shared with the scientific community. Thus, for scientific, ethical, and training reasons, it is important to understand the frequency and quality of dissertation publication in psychology.

There is an abundance of literature relevant to this topic, including student or faculty perspectives (e.g., [11–13]) and studies of general research productivity during doctoral training and early career periods (e.g., [14–19]). However, evidence specifically regarding dissertation publication is remarkably sparse and inconsistent [8,20–24]. This literature is limited by non-representative samples, biased response patterns, and disciplinary scopes that are either too narrow or too broad to offer insights that are useful and generalizable for psychological science. For example, in the only psychology-specific study to our knowledge, Porter and Wolfle [23] mailed surveys to a random sample of individuals who earned their psychology doctorates. Of 128 respondents, 59% reported that their dissertation research had led to at least one published article. Unfortunately, this study [23] and others (e.g., [8]) are now over 40 years old, offering little relevance to the present state of training and research in psychology. A much more recent and rigorous example comes from the field of social work. Using a literature searching methodology and a random sample of 593 doctoral dissertations in social work, Maynard et al [22] found that 28.8% had led to peer-reviewed publications. However, this estimate likely does not generalize to psychology and its myriad subfields. Thus, there is a need for more comprehensive, rigorous, and recent data to better understand dissertation publication in psychology.

Accordingly, the present study investigated the extent and nature of dissertation publication in psychology, specifically examining the following questions: (a) How many dissertations in psychology are eventually published in peer-reviewed journals? (b) How long does it take from dissertation approval to article publication? (c) What is the scientific impact of published dissertations (PDs)? and (d) Are there differences across subfields of psychology? Based on the literature and our own observations, we hypothesized that (a) a majority of dissertations in psychology would go unpublished; (b) dissertation publication would occur primarily during the first few years after Ph.D. approval, diminishing thereafter; (c) PDs would show evidence of at least moderate scientific influence via citation rates and journal metrics; and (d) professional/applied subfields (clinical, counseling, school/educational, industrial-organizational, behavioral) would yield fewer PDs than research-oriented subfields (social/personality, experimental, cognitive, neuroscience, developmental, quantitative).

## Materials and methods

### Sample

The dataset of psychology dissertations was obtained directly from ProQuest UMI’s *Dissertations and Theses* Database (PQDT), which is characterized as “the world’s most comprehensive collection of dissertations and theses. . . [including] full text for most of the dissertations added since 1997. . . . More than 70,000 new full text dissertations and theses are added to the database each year through dissertations publishing partnerships with 700 leading academic institutions worldwide” [25]. While international coverage varies across countries, PQDT’s repository is estimated to include approximately 97% of all U.S. doctoral dissertations [26], across all disciplines, institutions, and training models.

**Fig 1:**
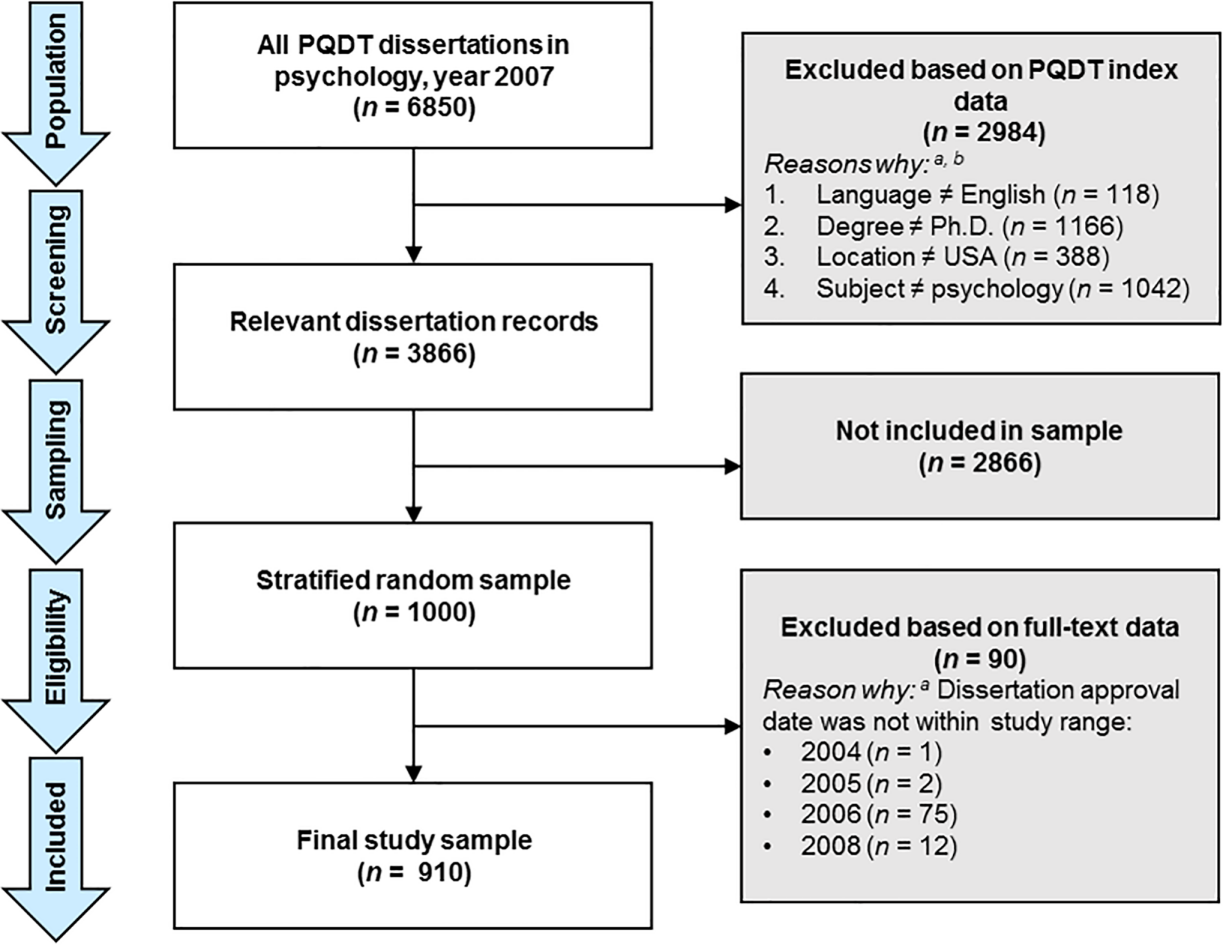
Flow diagram reflecting the numbers of dissertations included at each stage of the sampling process. Note. PQDT = ProQuest Dissertations and Theses. ^a^ Categories of excluded dissertations are mutually exclusive, summing to 100%. ^b^ PQDT exclusion criteria were applied sequentially in the order presented; thus, the number associated with each exclusion criterion reflects how many were excluded from the sample that remained after the previous criterion was applied. Adapted from the Preferred Reporting Items for Systematic Reviews and Meta-Analyses (PRISMA) flow diagram [27].

Upon request, PQDT provided a database of all dissertations indexed with the term “psychology” in the subject field during the year 2007. This resulted in a total population of 6,580 dissertations, which were then screened and sampled according to pre-defined criteria. The number of dissertations included at each stage in the sampling process is summarized in a PRISMA-style [27] flow diagram for the overall sample in Fig 1, and broken down by subfield in Table 1. Dissertations were excluded if written in a language other than English, for any degree other than Ph.D. (e.g., Psy.D., Ed.D.), or in any country other than U.S. The remaining dissertations were recoded for subfields based on the subject term classification in PQDT, with a few modifications (e.g., combining “neuroscience” and “biological psychology”). This left a remaining sample of 3,866 relevant dissertations, representing our population. This figure is approximately in line with the U.S. National Science Foundation’s Survey of Earned Doctorates [28] estimate that 3,276 research doctorates in psychology were granted during the year 2007, suggesting that PQDT could be slightly broader or more comprehensive in scope.

**Table 1:** Stratified study sample by subfield of psychology.

Subfield	*1*. *Screening*		*2*. *Sampling*			*3*. *Eligibility*		*4*. *Included*		Sampling Weight for Analyses
	Relevant Dissertations		Stratified Random Sample Drawn			Excluded		Final Study Sample		
	*n*	%	*n*	%	% of Relevant	*n*	% of Sample	*n*	%	
Clinical	1434	37.1	179	17.9	12.5	20	11.2	159	17.5	2.08
School/educational	415	10.7	91	9.1	21.9	7	7.7	84	9.2	1.19
Developmental	345	8.9	86	8.6	24.9	7	8.1	79	8.7	1.04
Social/personality	337	8.7	86	8.6	25.5	7	8.1	79	8.7	1.02
Cognitive	330	8.5	85	8.5	25.8	7	8.2	78	8.6	1.01
Industrial-org.	238	6.2	78	7.8	32.8	9	11.5	69	7.6	0.79
Neuroscience	212	5.5	75	7.5	35.4	6	8.0	69	7.6	0.73
Counseling	131	3.4	67	6.7	51.1	5	7.5	62	6.8	0.51
Behavioral	128	3.3	66	6.6	51.6	4	6.1	62	6.8	0.50
Experimental	117	3.0	65	6.5	55.6	5	7.7	60	6.6	0.47
Quantitative	101	2.6	63	6.3	62.4	6	9.5	57	6.3	0.42
General/misc.	78	2.0	59	5.9	75.6	7	11.9	52	5.7	0.34
TOTAL	3866	100	1000	100.0	25.9	90	9.0	910	100.0	

Note. “Relevant dissertations” refers to all PQDT dissertations that satisfied screening criteria for inclusion. Dissertations were excluded in the eligibility stage based on the date of approval in the full text (see Fig 1). Rates of exclusion were not significantly different across subfields. Sampling weights for each subfield were calculated as the proportion in the population (relevant dissertations) divided by proportion in the full sample, after adjusting for the proportions within each subfield that were from excluded for ineligibility.

From this relevant population of 3,866, we drew a stratified random sample of 1,000 dissertations. This number was selected because it represented over 25% of the population and offered sufficient power to obtain 95% CIs less than ±3% for the overall proportion estimates (i.e., the primary research question). As shown in Table 1, the sampling procedure was stratified by subfield using a formula that sought to balance (a) power for between-group comparisons, aiming to include ≥50 dissertations from each subfield; and (b) representativeness to the population, aiming to include ≥10% of the dissertations from each subfield. This resulted in subfield sample sizes ranging from 59 for general/miscellaneous (75.6% of relevant subfield population) to 179 for clinical (12.5% of relevant subfield population). Ninety (9.0%) dissertations were later found to be ineligible during the full-text review because the approval date was before or after the year 2007. This incongruence was partly explained by copyright or graduation dates differing from the dissertation year, and was not significantly different across subfields. The resulting final sample consisted of 910 dissertations, with subfield samples ranging from 52 (general/miscellaneous) to 159 (clinical). Because this study did not meet the definition of human subjects research, institutional review board approval was not required.

### Search timeframe

We aimed to conduct prospective follow-up searches for PDs within a timeframe that was both (a) long enough to capture nearly 100% of PDs and (b) short enough for results to retain their relevancy to the current state of psychological science. Because the literature does not offer dissertation-publication “lag time” statistics for reference, we used the “half-life” of knowledge—i.e., the average time it takes for half of a body of knowledge to become disproven or obsolete [29–30]. Across methodologies, the half-life of knowledge in psychology has been estimated at 7–9 years [31–33]. Accordingly, we selected a prospective search window allowing 0–7 years for dissertations to be published. Because the doctoral dissertations were sampled from the year 2007, follow-up searches were restricted to articles published between 2007 and 2014. We elected to exclude candidate publications from years prior to 2007 for several reasons. First, most U.S. psychology Ph.D. programs follow a more traditional dissertation model (and this would have been even more ubiquitous in 2007), where the dissertation would have to be completed before it could be published in a peer-reviewed journal. Second, even for the minority of programs that might follow less conventional models such as dissertation-by-publication [34], the lag-time to publication would likely still result in at least one PD appearing in print concurrently with or after the dissertation, and would therefore be captured by our search strategy. Finally, any potential benefits of searching retrospectively were outweighed by the potential risks of introducing unreliability into the data, such as identifying false positives from student publications, master’s theses, pilot studies, or other analyses from the same sample. On the other end of our search window, candidate publications that appeared in print during or after 2015 were also not considered. Post hoc analyses (see Results) suggested that this 0–7 year timeframe was adequate.

### Publication search and coding procedures

Searches for PDs were conducted in two rounds, utilizing scholarly databases in a manner consistent with the evidence regarding their specificity, sensitivity, and quality. Specifically, searches were conducted first in PsycINFO, which has high specificity for psychological, social, and health sciences [35–36]; and second, in Google Scholar, casting a much broader net but still searching for peer-reviewed scholarly journal articles [35,37–40]. The objective of these searches was to locate the PDs or to determine that the dissertation had *not* been published in the indexed peer-reviewed journals. Although it is never possible to definitively ascertain a thing’s non-existence, we added additional steps and redundancies to ensure that our searches were as exhaustive as possible. First, when no PD was found in either scholarly databases, as a final step we conducted Google searches for the dissertation author and title, then reviewed the search results (e.g., CVs posted online, faculty web pages) for possible PDs. Second, all searching/coding procedures were performed at least twice by trained research assistants. If two coders disagreed on whether a PD was found, which article it was, or if either coder was uncertain, these dissertations were then coded by consensus among three or more members of the research team, including master’s-level researchers (SCE and CMA).

In all literature searches, the following queries were entered for each dissertation: (a) title of dissertation, without punctuation or logical operand terms; (b) author/ student’s name; and (c) chair/ advisor’s name. Search results were assessed for characteristics of *authorship* (student and chair names), *content* (title, abstract, acknowledgments, methods), and *publication type* (specifically targeting peer-reviewed journal articles) by which a PD could be positively identified. Determination of PD status was made and later validated based on global judgments of these criteria. Identified PDs were then coded for their bibliographic characteristics. Results were excluded if published in a non-English journal, outside of the 0–7 year (2007–2014) window, or in a non-refereed or non-journal outlet (e.g., book chapters). Because dissertations can contain multiple studies and be published as multiple articles, searches aimed to identify a single article that was most representative of the dissertation, based on the criteria outlined above and by consensus agreement among coders. All searches were conducted and coding was completed between January 2015 and May 2017.

### Variables

#### Dissertation, publication, and year

Although the structure and content of doctoral dissertations varies across institutions, countries, and disciplines, the common unifying factor is that the dissertation represents an original research document produced by the student, approved by faculty, and for which a degree is conferred. Accordingly, in using PQDT as our population of U.S. Ph.D. psychology dissertations, we adhere to this broad but essential definition of a dissertation. This definition includes all different models of dissertations (e.g., ranging from traditional monographs to more recent models, such as briefer publication-ready dissertations and dissertation by publication [34]), but does not differentiate among them.

In this paper and in common scientific usage, “publication” refers to the dissemination of a written work to a broad audience, typically through a journal article. Accordingly, we do not consider indexing in digital databases for theses and dissertations as a publication such as in PQDT, even though it may be called “publishing” by the company. Rather, we define “dissertation publication” as the dissemination of at least part of one’s Ph.D. dissertation research in the form of an article published in a peer-reviewed journal. The peer-reviewed status of the journal was included among the variables that were coded twice with discrepancies resolved by consensus. Lastly, year of publication (2007, 2008, 2009 … 2014) and years since approval (0, 1, 2 … 7) were coded from when the print/final version of the article appeared, given that advance online access varies and is not available in all journals.

#### Subfield

The PQDT subject terms were used as a proxy indicator of the subfield of psychology from which the dissertation was generated. As described above, twelve categories were derived (Table 1). We considered five categories as *professional/ applied* subfields (clinical, counseling, educational/school, industrial-organizational, and behavioral), given that graduates in these fields are trained for careers that often include professional licensure or applied activities (e.g., consultation, program evaluation). In contrast, seven categories were considered *research/ academic subfields* (cognitive, developmental, experimental, neuroscience, quantitative, and social/personality), given that these subfields train primarily in a substantive or methodological research area. Note that Ph.D. programs in all of these subfields train their students to conduct research; when professional/ applied training components are present, they are there in addition to, not instead of, research training.

#### Article citations

The influence of PDs was estimated using article- and journal-level variables. At the article level, we used Web of Science to code the number of citations to the PD occurring each year since publication, tracking from 2007 up through year 2016. Importantly, Web of Science has been found to exhibit the lowest citation counts, but the citations which are included are drawn from a more rigorously controlled and higher quality collection of scholarly publications compared to others like Google Scholar, PubMed, and Scopus [35,37–38,40–41]. Citations were coded and analyzed primarily as the mean number of citations per year in order to account for time since publication. Total citations and citations each year were also calculated.

#### Journal-level metrics

The following journal impact metrics were recorded for the year in which the PD was published: (a) Impact Factor (IF) and (b) 5-Year IF [42]; (c) Article Influence Score (AIS) [43]; (d) Source Normalized Impact (SNIP) [44]; and (e) SCImago Journal Rank indicator (SJR) [45]. Each of these indices shares different similarities and distinctions from the others and provides different information about how researchers cite articles in a given journal. While each has its limitations, these five indicators together offer a broad overall characterization of a journal’s influence, without over-relying on any single metric. As a frame of reference, the population-level descriptive statistics for each of these journal metrics (2007–2014) are as follows: IF (*M* = 1.8, *SD* = 2.9), 5-year IF (*M* = 2.2, *SD* = 3.0), SNIP (*M* = 0.9, *SD* = 1.0), SJR (*M* = 0.6, *SD* = 1.1), and AIS (*M* = 0.8, *SD* = 1.4).

As described above, all of the dissertation, literature searching, and outcome data used in the present study were obtained from a variety of online sources available freely or by institutional subscription. Links to these sources can be found in the supplementary materials (S1 File).

### Analytic plan

Overall descriptive analyses were conducted to examine the univariate and bivariate characteristics of the data, including the frequency and temporal distribution of PDs in psychology. Similar descriptive statistics were provided to characterize the nature of and scholarly influence of the PD via article citations and journal impact metrics. Group-based analyses were conducted using chi-square and ANOVAs to assess whether dissertation publication rates and scientific influence differed across subfields of psychology. The 95% CIs surrounding the total weighted estimate were used as an index of whether subfield estimates were significantly above or below average.

Time-to-publication analyses were conducted in three different ways. First, we used weighted Cox regression and Kaplan-Meier survival analyses to model dissertation publication as a time-to-event outcome, both for the overall sample and separately by subfields. Second, because the large majority of dissertations “survived” the publication outcome past our observation window (i.e., most cases were right-censored), we also conducted between-group comparisons regarding subfield publication times for only those whose dissertations were published. Finally, in order to ensure the adequacy of our 0–7 year search window, we fit a distribution to our observed data and projected this trend several years into the future.

Full-sample analyses were conducted using the complex samples option in SPSS Version 24, which yields weighted estimates that are less biased by sample proportions and more generalizable to the population. Distribution model-fitting and projections were estimated in R. For analyses related to dissertation publication outcomes, there were no missing data because all values could be coded based on obtained dissertations. Data availability for journal- and article-level variables are reported in those results tables.

## Results

### Frequency of and time to publication

The overall weighted estimate showed that 25.6% (95% CI: 23.0, 28.4) of psychology dissertations were published in peer-reviewed journals within the period of 0–7 years following their completion. The unweighted estimate was similar (27.5% [24.6, 30.4]), but reflected sampling bias due to differences between subfields. Thus, weighted estimates are used in all subsequent results. Significant variations were found across subfields (Rao-Scott adjusted χ^2^(*df* = 9.65) = 65.28, *F*(9.65, 8869.62) = 8.28, *p* < .001). As shown in Table 2, greater proportions of PDs were found in neuroscience (59.4% [47.8, 70.1]), experimental (50.0% [37.7, 62.3]), and cognitive (41.0% [31.1, 51.8]), whereas much lower rates were found for industrial-organizational (10.1% [5.0, 19.5]) and general/miscellaneous (13.5% [6.5, 25.7]). All other subfields fell between 19.0 and 29.0%. Quantitative and social/personality fell within the 95% CIs for the weighted total, suggesting no difference; however, most other subfields fell above or below this average. Of note, three core professional subfields (clinical, counseling, and school/educational) were all between 19.0 and 20.8%—below average and not different from one another.

**Table 2:** Percentage of dissertations published.

Subfield	Subfield Rank	Estimate (%)	95% CI	
			LB	UB
Behavioral	4^a^	29.0	19.2	41.3
Clinical	8^b^	20.8	16.2	26.3
Cognitive	3^a^	41.0	31.1	51.8
Counseling	9^b^	19.4	11.4	31.0
Developmental	7^b^	21.5	14.1	31.5
Experimental	2^a^	50.0	37.7	62.3
General/misc.	11^b^	13.5	6.5	25.7
Industrial-org.	12^b^	10.1	5.0	19.5
Neuroscience	1^a^	59.4	47.8	70.1
Quantitative	5	28.1	18.0	41.0
School/educational	10^b^	19.0	12.3	28.3
Social/personality	6	26.6	18.3	36.9
Total		25.6	23.0	28.4

^a^Greater than weighted total

^b^Less than weighted total.

**Fig 2:**
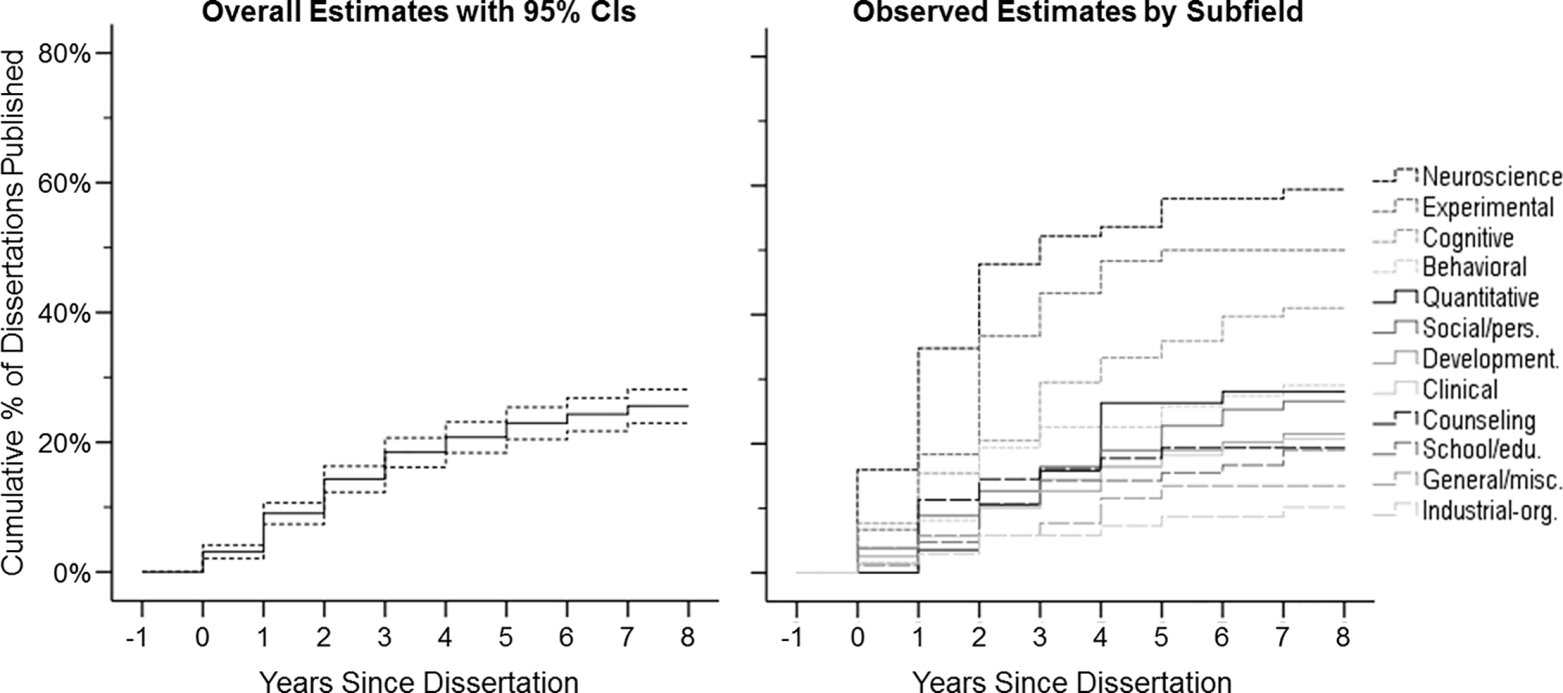
Estimated cumulative rates of dissertation publication over time. Overall estimates and 95% confidence intervals (left panel) are derived from the weighted Cox regression model (see Table 3). Subfield estimates (right panel) are derived from the unweighted Kaplan-Meier regression model. In both plots, cumulative publication estimate = one minus survival function.

The overall time-to-publication results are presented in Table 3 and the left panel of Fig 2. As shown, over half (56.0% of those ultimately published; 14.3% of the total sample) of PDs appeared in print within 2 years following the year of completion, with the large majority (89.7% of ultimately published; 23.0% of total) being published within 5 years. Among those dissertations that were ultimately published, the time to publication averaged about 2–3 years (*M* = 2.58 [2.34, 2.83]), with a median of 2 years and a mode of 1 year. Omnibus comparisons from the Kaplan-Meier survival model revealed significant variations across subfields, χ^2^(*df* = 1) = 4.24, *p* = .039), as plotted in the right panel of Fig 2. These results generally mirrored the same pattern found for overall binary publication outcomes across subfields. Among only those dissertations that were published, the subfield differences in time-to-publication were marginal overall, *F*(11, 238) = 5.99, *p* = .064, but still shed some additional light beyond the binary publication outcomes. Specifically, neuroscience (*M* = 1.61, [1.09, 2.13]), counseling (*M* = 1.92, [1.04, 2.79]), and experimental (*M* = 1.93 [1.45, 2.41]) averaged less than two years to publication, shorter than the weighted average. In contrast, clinical (*M* = 2.88, [2.20, 3.56]), social/personality (*M* = 2.90, [1.92, 3.89]), school/educational (*M* = 2.94, [1.78, 4.1]), industrial-organizational (*M* = 3.00, [0.73, 5.27]), and quantitative (*M* = 3.06, [2.35, 3.78]) all took longer, approximately three years.

**Table 3:** Temporal lag from dissertation completion to publication by year.

Years after Dissertation	% Dissertations Published	Cumulative Estimate	Cumulative 95% CI	
			LB	UB
0	3.1	3.1	2.1	4.1
1	5.9	9.0	7.4	10.7
2	5.3	14.3	12.3	16.3
3	4.1	18.5	16.2	20.7
4	2.3	20.8	18.4	23.1
5	2.2	23.0	20.4	25.4
6	1.4	24.3	21.7	26.8
7	1.3	25.6	22.9	28.1

Note. Cumulative estimates and 95% confidence intervals are derived from the weighted Cox regression model, where cumulative publication estimate = one minus survival function. All estimates based on the full sample (denominator *n* = 910 for all percentages). See Fig 2, left panel, for a visual presentation of these results.

Lastly, as a methodological check, we modeled our time-to-publication data and projected this trend into the future to estimate what percentage of PDs we might have missed by stopping after 7 years. More specifically, these models used the weighted estimates of how many dissertations were published each year as the outcome and time (years 0 to 7) as the predictor. A Poisson model containing quadratic and linear effects for time fit the data best. When projected into the future, this model estimated that an additional 7 dissertations would be published at 8–10 years post-dissertation (4, 2, and 1 PDs, respectively). From 11 years onward, estimates asymptotically approached and rounded down to zero, even cumulatively. Thus, our sampling frame appears to have captured virtually all (97.3%) of the dissertations that ultimately would be published. In other words, had the study been implemented for as long as necessary to capture *all* PDs, the data suggest that our primary result, the estimated percentage of dissertations published, would increase only modestly from 25.6% to 26.4%.

### Scientific impact

As shown in Table 4, PDs were cited an average of 3.65 times per year since publication, totaling 15.95 citations on average during the years captured by the study. There were significant variations by subfield in terms of both total and per-year citations. Specifically, PDs in cognitive (*M* = 5.08 [1.33, 8.83]) and industrial-organizational (*M* = 5.18 [0.80, 9.56]) were more highly cited, with over 5 citations/year. On the other end, fields that exhibited relatively lower (but still nontrivial) rates of citations/year included quantitative (*M* = 1.42, [0.87, 1.97]), general/miscellaneous (*M* = 1.46 [0.15, 2.78]), counseling (*M* = 1.64 [0.63, 2.64]), developmental (*M* = 2.82 [1.31, 4.32]), and social/personality (*M* = 2.86 [2.05, 3.67]).

**Table 4:** Cumulative article citations in Web of Science per year after publication.

Years After Publication	Data Availability (*n*)	Mean			Range		Subfield differences		
		Est	95% CI		Min	Max	Wald *F*	*df*	*p*
			LB	UB					
0	211	0.37	0.26	0.48	0	5	—	—	—
1	211	2.09	1.73	2.46	0	22	—	—	—
2	211	5.19	4.31	6.07	0	55	—	—	—
3	204	9.20	7.55	10.86	0	112	—	—	—
4	198	13.33	10.89	15.78	0	155	—	—	—
5	182	18.39	14.69	22.09	0	221	—	—	—
6	160	24.02	18.65	29.40	0	301	—	—	—
7	126	30.23	22.08	38.37	0	372	—	—	—
Per year	211	3.65	2.88	4.42	0	57	2.80	11,189	.002
Total	211	25.95	19.71	32.20	0	456	3.32	11,189	< .001

Note. Estimates are calculated from available years from the publication year (inclusive) through 2016. This sampling range naturally truncates the number of follow-up years available for dissertations published in later years (e.g., 7-year outcomes are available only for dissertations published in 2007 to 2009, *n* = 126).

The 250 PDs in our sample appeared in 186 different peer-reviewed outlets, including top-tier journals in general (e.g., *Nature*, *Science*) and psychological (e.g., *Psychological Science*, *Journal of Consulting and Clinical Psychology*) science. Notably, several PDs appeared in journals predominately representing professions or disciplines outside psychology (e.g., *Public Health Nursing*, *Endocrinology*). The most common journal titles were all in relatively specialized areas of psychology (e.g., *Applied Psychological Measurement*, *Brain Research*), tending to draw from experimental, social/personality, neuroscience, behavioral, and cognitive. Overall, however, dissertations were disseminated broadly, with no single journal “catching” more than five (2.0%) dissertations from our overall sample, and most journals publishing only one (0.4%).

As shown in Table 5, PDs appeared in journals of moderate-to-high influence according to all five metrics used. Subfield differences were found for the IF, SNIP, and SJR (*p*s < .01, but not in the 5-year IF or the AIS (*p*s > .09). Specifically, neuroscience and cognitive PDs appeared in higher-IF journals (*M*s = 4.47 [3.17, 5.78] and 3.86 [1.87, 5.86], respectively), while most others fell in the below-average IF, including those still within the 2+ range (clinical, social/personality, general/miscellaneous, developmental, and behavioral; *M*s = 2.14 to 2.45) and those in the 1–2 range (quantitative, school/educational, counseling, and industrial-organizational; *M*s = 1.27 to 1.71). Similarly, neuroscience (*M* = 2.17 [1.68, 2.66]), cognitive (*M* = 1.97 [1.22, 2.72]), and social/personality (*M* = 1.65 [1.09, 2.21]) PDs appeared in higher-SJR journals, whereas behavioral, clinical, general/miscellaneous, quantitative, school/educational, industrial-organizational, and counseling PDs had lower SJRs (*M*s = 0.51–1.21). Lastly, cognitive (*M* = 1.61 [1.17, 2.05]) and social/personality (*M* = 1.55 [1.18, 1.92]) were published in higher-SNIP journals, while clinical (*M* = 1.28 [1.08, 1.47]), general/miscellaneous (*M* = 1.19 [0.23, 2.15]), and counseling (*M* = 0.57 [0.26, 0.89]) PDs appeared in journals with lower SNIPs.

**Table 5:** Journal-level impact metrics.

Metric	Data Availability (*n*)	Mean			Range		Subfield Differences		
		Estimate	95% CI		Min	Max	Wald *F*	*df*	*p*
			LB	UB					
IF	209	2.84	2.45	3.23	0.20	31.43	2.92	11,187	.001
5yr IF	203	3.49	3.07	3.90	0.29	31.21	1.57	11,181	.110
AIS	203	1.36	1.14	1.58	0.13	17.28	1.63	11,181	.093
SJR	231	1.46	1.36	1.62	0.11	11.08	3.80	11,209	< .001
SNIP	232	1.39	1.28	1.50	0.07	7.56	2.44	11,210	.007

## Discussion

The primary finding of this study was that only about one in four psychology Ph.D. dissertations in the U.S. was published in a peer-reviewed journal. Typically this occurred within 2–3 years after completing the dissertation. Despite variations across subfields, dissertation publication appears to be the exception not the rule. When dissertations were published, however, they were often highly cited and appeared in influential journals. The relatively high impact of published dissertations may reflect a gatekeeping effect, whereby only the highest quality or most significant contributions get published; or a refining effect, whereby the dissertation development and committee review process helps strengthen the contribution [1,46], increasing the likelihood and impact of publication. In other words, the dissertation process may add some value to doctoral research, and some doctoral research appears to add value to psychological science. A larger and more important question is why the vast majority of psychology dissertation research does not contribute to the peer-reviewed literature.

Our estimated rate of dissertation publication in psychology (25.6%) is similar to or slightly below a corresponding estimate for social work (28.8%) [22], the only field in which a similarly rigorous and comparable design had been used. To our knowledge, the present study is the first to offer a reliable estimate of publication rates specific to the dissertation and specific to psychology. Further, the present study advances the literature by demonstrating the impact that these published dissertations have on the scientific literature. Although it was only minority of cases, published dissertations in psychology were disseminated in moderate- to high-impact journals across a wide spectrum of disciplines and specialty interests. Whereas published dissertation articles were cited several times per year, anecdotally we saw very few citations to the actual dissertation documents in PQDT. These observations are consistent with evidence showing that the impact of dissertations themselves has declined markedly [7–8] in recent decades. In contrast, peer-reviewed journal articles are much more likely to be read, cited, and included in systematic and meta-analytic reviews.

Subfield differences were broadly consistent with hypotheses. Dissertations from professional/applied fields were less often published, whereas the more research/academic-oriented subfields published at rates much higher than average. These findings likely reflect differences in the nature of training and motivation in professional and scientific subfields, and also align with evidence about student research productivity in professional/applied subfields. For example, annual results from the Association of Psychology Postdoctoral and Internship Centers applicant survey indicate that only about 50% of advanced doctoral students in professional psychology have authored or co-authored any peer-reviewed publications, while only 10% have published 5 or more [18]. Given this relatively low baseline rate of productivity during graduate school for this population, the average likelihood of post-graduation publication seems low. On the other hand, individuals in more research-oriented subfields are often training specifically for an academic position which incentivizes publication. Further, lab-based dissertations often include multiple experiments, which may create more publishable units (this also may explain the relatively higher rate of publication in behavioral psychology). The low publication of dissertations in industrial-organizational (10%) is also interesting, and may reflect an applied focus, organizational propriety of data, greater non-academic incentives (e.g., higher salaries in industry), or limited generalizability as market or consultative research. When these and other types of applied/professional dissertations were published, however, they were often cited several times per year.

The time from dissertation completion to publication appears to be a critical consideration. From our main results and longitudinal projections, we can generalize that by two calendar-years post-dissertation, over 50% of ultimately-published dissertations will appear in print. After five years, this number increases to nearly 90% (10% probability of publication). After 7–10 years, the dissertation findings are likely to become outdated, irrelevant, or overturned [30–32], and the probability of publication approaches 0%. Thus, if students wish to publish their dissertation, it is recommended that they proactively develop a plan for adapting the full document into a manuscript (or multiple manuscripts) for publication [1]. As one example of this, we are aware of some universities that have begun requiring that approved dissertations be accompanied by a form that outlines an agreed-upon plan for publication and authorship.

The present findings raise questions about the reasons for nonpublication. Possible explanations include the burden of revising and submitting a lengthy document, or limited career incentives for pursuing publications in non-academic careers. Alternatively, unpublished dissertations may lack methodological rigor, including “fatal flaws,” or fail to make a novel and substantive contribution. Thus, unpublished dissertations might not pass the bar of peer review. The present results only illustrate how many dissertations were actually published, and cannot speak to how many students attempted to publish their dissertations, or how many dissertations might have been publishable quality. Similarly, these results do not provide direct evidence of the mechanisms underlying publication vs. nonpublication, but the apparently high quality of the published dissertation articles is consistent with the file drawer hypothesis. Interestingly, one recent study in management research found that in the path from dissertation to publication, studies appear to get “beautified,” for example, such that the ratio of supported to unsupported hypotheses more than doubles in at least one discipline [47]. Such questionable research practices may provide one explanation for how dissertations selectively get published, but this is clearly not an appropriate solution. Whatever the underlying explanations may be, the widespread non-publication of dissertation research is a problem in psychology. To the extent that this non-publication continues, it exacerbates the file drawer problem [5–6,9], biases systematic reviews and meta-analyses, and contributes to the replication problem in psychology [10]. It also amounts to inefficient use of time and resources, raising ethical questions about violating agreements with participants and funding agencies, and about the consequences of not disseminating research findings [2,4].

The present study was designed so that results could be generalized to the population of dissertations produced in U.S. psychology Ph.D. programs. However, some limitations should be noted. First, our stratified random sample was drawn from an archival data source (PQDT), which is an approximation of the population of dissertations in psychology (although a very comprehensive one) and a proxy of the boundaries delineating subfields in psychology. Our outcome variables were likewise drawn from various databases (e.g., PsycINFO, Google Scholar, Web of Science, Thomson Reuters) which are necessarily restricted in different ways. As noted in the Methods section, these databases were selected as the most comprehensive and appropriate sources available for the purposes for which they were used, and their strengths and weaknesses were considered in developing the study protocol.

A second constraint lies in the selection of a single cohort year (2007) and 7-year follow-up period, raising the possibilities of missed cases and of cohort/historical effects. The changing landscape of doctoral training in psychology (e.g., more competitive admissions, increasing emphasis on research productivity, nontraditional dissertation requirements) may limit generalizability to past and future populations. Of note, these results may not generalize broadly to other countries (e.g., Northern/Western Europe, Australia, and New Zealand) and fields (e.g., biomedical, natural, and physical sciences) that are increasingly using a dissertation-by-publication model [34]. From our read of the literature and our assessment of the present sample, this model has not been widely adopted in U.S. psychology, where more traditional dissertation documents are still the norm. Accordingly, our sampling and search strategies were designed to work reasonably well for all U.S. psychology dissertations, including a suspected minority of nontraditional models; however, we could not differentiate types of dissertations. For all of these reasons, periodic replication of these results would be useful. Nonetheless, the large sample size, stratified sampling method, comprehensive dataset, and thorough multi-stage search protocol help mitigate bias. Narrow 95% confidence intervals support the precision of the overall estimate, and post hoc analyses suggested that the results are unlikely to change substantially given a longer sampling frame.

Finally, we note that this study should not be interpreted as any sort of evaluation of students, advisors, or programs for dissertations that were or were not published. Nor are we advocating that all dissertations be published, regardless of quality. Rather, these findings shed light on what appears to be a systemic problem affecting research and training in all areas of psychology. Efforts aimed at increasing the quality and “publishability” of doctoral dissertation research may have broad benefits for both training and research in psychology. On the training side, these efforts may benefit students and graduates in terms of providing a higher standard of scientific training, more research/publishing experience, and greater early-career productivity. On the research side, such efforts can help promote a higher level of rigor in doctoral research and increase the likelihood that the findings will be shared with the scientific community.

## Supporting information

S1 File Links to data sources. (PDF)
