# Supplementary material for: "Are you gonna publish that?" Peer-reviewed publication outcomes of doctoral dissertations in psychology
Source: PLoS One. 2018 Feb 14;13(2):e0192219. doi: 10.1371/journal.pone.0192219 (PMC5812605; doi:10.1371/journal.pone.0192219)
Supplement: S1 File — (PDF) [file pone.0192219.s001.pdf]

Source	Data Obtained	URL
ProQuest Dissertations & Theses	• Dissertation sample<br>• Subfield classifications	http://www.proquest.com/products-services/pqdtglobal.html
PsycINFO	• Peer-reviewed articles derived from dissertations (focused search)	http://www.apa.org/pubs/databases/psycinfo/index.aspx
Google Scholar	• Peer-reviewed articles derived from dissertations (broad search)	https://scholar.google.com/
Web of Science	• Article citations by year<br>• Thomson Reuters Journal Impact Factor<br>• Thomson Reuters 5-Year Impact Factor	https://clarivate.com/products/web-of-science/
Scopus Journal Metrics	• Article Influence Score<br>• Source Normalized Impact per Paper<br>• SCImago Journal Rank indicator	https://journalmetrics.scopus.com/
